# Projected northward shifts in eastern red‐backed salamanders due to changing climate

**DOI:** 10.1002/ece3.9999

**Published:** 2023-04-26

**Authors:** Brandon P. Hedrick, Alba Estrada, Chris Sutherland, A. Márcia Barbosa

**Affiliations:** ^1^ Department of Biomedical Sciences Cornell University Ithaca New York USA; ^2^ Pyrenean Institute of Ecology (CSIC) Jaca Spain; ^3^ Centre for Research into Ecological and Environmental Modelling University of St Andrews St Andrews UK; ^4^ Centro de Investigação em Ciências Geo‐Espaciais Vila Nova de Gaia Portugal

**Keywords:** conservation biogeography, ecological niche models, favorability, fuzzy logic, *Plethodon*, Plethodontidae, species distribution models

## Abstract

Many species' distributions are being impacted by the acceleration of climate change. Amphibians in particular serve numerous ecosystem functions and are useful indicators of environmental change. Understanding how their distributions have been impacted by climate change and will continue to be impacted is thus important to overall ecosystem health. *Plethodon cinereus* (Eastern Red‐Backed Salamander) is a widespread species of lungless salamander (Plethodontidae) that ranges across northeastern North America. To better understand future potential lungless salamander range shifts, we quantify environmental favorability, the likelihood of membership in a set of sites where environmental conditions are favorable for a species, for *P. cinereus* in multiple time periods, and examine shifts in the species' distribution. First, utilizing a large data set of georeferenced records, we assessed which bioclimatic variables were associated with environmental favorability in *P. cinereus*. We then used species distribution modeling for two time periods (1961–1980 and 2001–2020) to determine whether there was a regional shift in environmental favorability in the past 60 years. Models were then used to project future distributions under eight climate change scenarios to quantify potential range shifts. Shifts were assessed using fuzzy logic, avoiding thresholds that oversimplify model predictions into artificial binary outputs. We found that *P. cinereus* presence is strongly associated with environmental stability. There has been a substantial northward shift in environmental favorability for *P. cinereus* between 1961–1980 and 2001–2020. This shift is predicted to continue by 2070, with larger shifts under higher greenhouse gas emission scenarios. As climate change accelerates, it is differentially impacting species but has especially strong impacts on dispersal‐limited species. Our results show substantial northward shifts in climatic favorability in the last 60 years for *P. cinereus*, which are likely to be exacerbated by ongoing climate change. Since *P. cinereus* is dispersal‐limited, these models may imply local extirpations along the southern modern range with limited northward dispersal. Continued monitoring of amphibians in the field will reveal microclimatic effects associated with climate change and the accuracy of the model predictions presented here.

## Graphical abstract

As climate change accelerates, it is differentially impacting species but has especially strong impacts on species that are dispersal limited. Our results show substantial northward shifts in environmental favorability for red‐backed salamanders in the last 60 years, which are likely to be exacerbated by ongoing climate change.

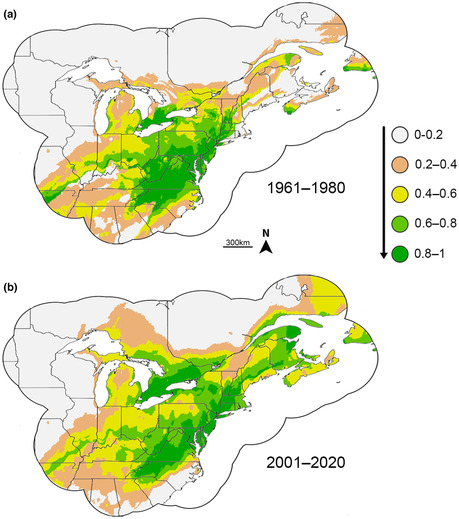

## 1 INTRODUCTION

Anthropogenic climate change has been accelerating and will continue to accelerate over the coming decades, which will have profound impacts on both human and natural systems (Claeys et al., 2019; Nerem et al., 2018; Smith et al., 2015). Changes in a wide variety of variables such as temperature and precipitation (e.g., Stendel et al., 2021) have already impacted species ranges for many taxa across the world (e.g., Aragón et al., 2010; Báez et al., 2020; Hill et al., 2011). The capacity of a species to shift its range in response to environmental disturbance or climatic changes grants species the ability to persist over long time scales. For example, nonmigratory European butterflies were shown to shift northward between 35 and 240 km in the 20th century (Parmesan et al., 1999), and coastal pelagic fishes have shifted poleward by hundreds of kilometers in the past 20 years (Champion et al., 2021) in response to climate and environmental changes. However, given the acceleration of climatic shifts, as well as other factors such as land‐use changes and urbanization, species may not be able to shift their ranges quickly enough and may suffer local extirpations. It is important to note that species are not all impacted equally (Estrada et al., 2015; Estrada, Morales‐Castilla, et al., 2016) with dispersal‐limited species being especially vulnerable (Enriquez‐Urzelai, Bernardo, et al., 2019; Muñoz, Miller Hesed, et al., 2016). Therefore, a better understanding of current and future range shifts is critically important to conservation initiatives.

Amphibians are highly sensitive to environmental conditions (Buckley & Jetz, 2007; Feder & Burggren, 1992), especially plethodontid (lungless) salamanders, which breathe entirely through their skin and prefer high moisture levels (Grover, 1998; Spotila, 1972), and warmer temperatures (McCarthy et al., 2017; Muñoz, Miller Hesed, et al., 2016; Peterman & Semlitsch, 2013). For example, *Hydromantes* (Plethodontidae) activity has been shown to be especially tied to specific temperature and precipitation‐based environmental conditions (Lunghi et al., 2016; Lunghi, Manenti, et al., 2018). Given their sensitivity to environmental change, accelerated climate change is likely to have profound impacts on amphibian distributions, some of which also have limited dispersal capabilities (Carey & Alexander, 2003; Donnelly & Crump, 1998; Muñoz, Miller Hesed, et al., 2016).

Among plethodontid salamanders, the eastern red‐backed salamander (*Plethodon cinereus*) has been extensively studied behaviorally (Fleming et al., 2021; Hedrick et al., 2021; Hernández‐Pacheco et al., 2019; Muñoz, Miller, et al., 2016; Muñoz, Miller Hesed, et al., 2016; Sanchez et al., 2020) and has a large range that spans across the northeastern part of the United States and southeastern Canada (Petranka, 1998). Throughout their range, they are a prevalent, yet cryptic element in forest ecosystems (Welsh & Droege, 2001). This previous work has established the microclimatic preferences of *P. cinereus*, such as how air and soil temperature and precipitation impact detection rates of *P. cinereus* (Hernández‐Pacheco et al., 2019), and how temperature impacts the species' growth rates and the onset of sexual maturity (Muñoz, Miller Hesed, et al., 2016). As a result of this wealth of research, *P. cinereus* has become a model species for understanding the impacts of microclimate on amphibian ecology, behavior, and dispersal (reviewed in Fisher‐Reid et al., 2021). However, the effects of macroclimate on *P. cinereus* have been less fully explored, and the potential impacts of future climate on the species as a whole have yet to be studied via species distribution modeling, which may provide important insights into potential distributional changes over the next century (e.g., Zhang et al., 2020 in Chinese giant salamanders).

Dispersal‐limited species are particularly vulnerable to climatic shifts, as rates of change are likely to be higher than the species' capacity to move to a more suitable habitat and expand its range (Estrada et al., 2015; Gibbons et al., 2000; Midgley et al., 2006; Muñoz, Miller Hesed, et al., 2016; Schneider & Root, 1998; but see Smith & Green, 2005). *P. cinereus* is dispersal limited and will likely need to respond to changing environmental conditions via alternative pathways such as phenotypic plasticity or vertical movement in the earth (rather than a horizontal movement to more suitable surface environments) (Muñoz, Miller Hesed, et al., 2016). Regardless, *Plethodon* species must come to the surface to feed and lay eggs (Petranka, 1998) and therefore the shifting surface conditions resulting from rapid climate change will likely have substantial impacts on *P. cinereus*' range and distribution.

Conserving species requires protecting the habitat within their distributions (Lomolino, 2004; Real et al., 2009). Species distribution models (SDMs) and ecological niche models are useful tools for understanding how a species' range may overlap with those of other species (Caravaggi et al., 2017; Real et al., 2009), how it has changed in the past (Gutiérrez‐Rodríguez et al., 2017a, 2017b; Warwick et al., 2021), and how it may change in the future under different climate projections (Báez et al., 2020; Pearson & Dawson, 2003; Real et al., 2010; Zhang et al., 2020), which may potentially lead to future species range expansions and contractions. SDMs have been used extensively to assess climate impacts on amphibian ranges (Barbosa & Real, 2012; Gutiérrez‐Rodríguez et al., 2017a, 2017b; Reino et al., 2017; Sánchez‐Montes et al., 2019), and it has been found that ectotherm distributions are more strongly and directly impacted by climactic changes, likely as a result of physiological constraints, compared with endotherms, which are more affected by indirect climate effects (Aragón et al., 2010).

There are a wide variety of procedures to perform SDMs, one of them being the favorability function (Real et al., 2006), which assesses how the local presence probability differs from that expected by chance, due to the local environmental conditions, independently of the species' prevalence. Environmental favorability has the advantage of being commensurable and thus directly comparable across species and time periods (Acevedo & Real, 2012; Barbosa, 2015; Real et al., 2006), allowing the use of fuzzy logic to directly combine continuous predictions without applying thresholds. We aim to determine the macroclimatic factors that affect the range of *P. cinereus*, and how future climate change may impact *P. cinereus* by assessing past, present, and future environmental favorability across its range. These data will generate new insights into large‐scale climatic impacts on *P. cinereus* and build on our current understanding of *P. cinereus* ecology. As *P. cinereus* is becoming a model system in amphibian ecology (Fisher‐Reid et al., 2021), these data will be potentially helpful in better understanding range shift ecology in amphibians generally, especially lungless amphibians, in the face of rapidly accelerating climate change.

To address our goal of understanding how the distribution of *P. cinereus* may be impacted by future climate change, we first evaluated the bioclimatic factors related to the modern *P. cinereus* distribution and whether the climatic favorability for *P. cinereus* has shifted spatially between two time periods: 1961–1980 and 2001–2020. Then, utilizing two climate models (CCSM4, MIROC‐ESM), each under four different greenhouse gas (GHG) emission scenarios, we used fuzzy logic to assess how changes in environmental favorability may impact the *P. cinereus* distribution by 2070. Specifically, we aim to (1) define the regions that are currently climatically favorable for *P. cinereus* across its range, based on SDMs built from data from the past 20 years (2001–2020) and determine which bioclimatic variables are influential in defining that range, (2) examine whether environmental favorability for *P. cinereus* has shifted in the last 60 years (with data from two time periods: 1961–1980 and 2001–2020), and (3) predict future range shifts and identify areas of projected expansions and contractions as a function of projected future climate. Based on previous data, we expect that *P. cinereus'* distribution will be strongly linked to precipitation‐based climatic variables, as has been found more generally for amphibians (Aragón et al., 2010; Buckley & Jetz, 2007; Lunghi, Manenti, et al., 2018). We further hypothesize that environmental favorability for *P. cinereus* will shift increasingly toward the north by 2070 under progressively higher GHG emission scenarios in both climate models. Finally, we hypothesize that the absolute amount of favorable area will decrease with increasingly higher GHG emissions.

## 2 METHODS

### 2.1 Data collection and climate variables

*Plethodon cinereus* occurrence data were downloaded from Global Biodiversity Information Facility (GBIF, 2021). This returned 106,424 spatially referenced records for *P. cinereus* as of September 2021 (https://doi.org/10.15468/dl.d3j5ys). Data were then checked for errors so that only suitable occurrence data were used in analyses. This involved removing all duplicates, retaining only records of confirmed presences, and using the *scrubr* v. 0.4.0. package in R (Chamberlain, 2020), removing occurrence records where the latitude–longitude data were incomplete, not possible, or unlikely (e.g., at 0, 0 coordinates). Finally, given that records of *P. cinereus* should only occur in the United States and Canada, only records within those countries were accepted. Several records were found in western North America that were not removed earlier in the process and thus they were removed by not accepting any occurrence records west of a decimal longitude of −94 or south of a decimal latitude of 32. This resulted in 102,697 records. Data were not evenly distributed through time (Figure S1a). The oldest record is from 1809, with the majority of records occurring before 1980 (79%) and after 2010 (only 7.1% of the records occurred between 1980 and 2010).

For evaluating shifts, we used subsets of the data for the periods 1961–1980 and 2001–2020. The 1961–1980 data subset contained 58,621 records (Figure S1b) and the 2001–2020 subset contained 12,521 records (Figure S1c). Occurrence data were then formatted to be organized in a spatial data frame. A buffer of 350 km around the occurrence data was then established using the buffer function in the *raster* v. 3.5‐29 R package (Hijmans, 2021) using the entire data set to establish environments that were accessible but where *P. cinereus* did not occur (Figure S2). The buffered region was used as the study area. One method for delimiting a study area is to use geographical barriers that the species is not capable of crossing. However, given the wide range of *P. cinereus*, we opted to have a buffered region that included inhospitable northern and southern climates as well as western climates where the species does not occur, but that are theoretically accessible to *P. cinereus*.

Environmental variables were downloaded from WorldClim (Hijmans et al., 2005) using the *sdmpredictors* v. 0.2.12 R package (Bosch, 2020) and then clipped to fit the buffered region outlined above. Each variable map had a spatial resolution of 0.08333 geographic degrees. The WorldClim variables included altitude, as well as bioclimatic variables (BIO1‐Annual Mean Temperature, BIO2‐Mean Diurnal Range, BIO4‐Temperature Seasonality, BIO5‐Maximum Temperature of Warmest Month, BIO6‐Minimum Temperature of Coldest Month, BIO7‐Temperature Annual Range, BIO8‐Mean Temperature of Wettest Quarter, BIO9‐Mean Temperature of Driest Quarter, BIO10‐Mean Temperature of Warmest Quarter, BIO11‐Mean Temperature of Coldest Quarter, BIO12‐Annual Precipitation, BIO13‐Precipitation of Wettest Month, BIO16‐Precipitation of Wettest Quarter, BIO17‐Precipitation of Driest Quarter, BIO18‐Precipitation of Warmest Quarter, BIO19‐Precipitation of Coldest Quarter) (Table 1). We excluded BIO3 (Isothermality), BIO14 (Precipitation of Driest Month), and BIO15 (Precipitation Seasonality) in our analyses due to a documented low correlation between present and future variables (Bedia et al., 2013). Occurrence data were then thinned with the gridRecords function of *fuzzySim* v. 4.3 package (Barbosa, 2015), to match the resolution of the climate variables. Pixels that were not intersected by any presence points were used as unoccupied background.

**TABLE 1:** Bioclimatic and altitude variables used to model *Plethodon cinereus* probability of presence and favorability. Variables were split into general categories (topography, climatic variability, environmental energy, and water availability).

Factor	Variable	Code
Topography	Altitude (m)	ALT
Climatic variability	Temperature seasonality (°C)	BIO4
Environmental energy	Annual mean temperature (°C)	BIO1
	Mean diurnal range (°C)	BIO2
	Max. temp. warmest month (°C)	BIO5
	Min. temp. coldest month (°C)	BIO6
	Temperature annual range (°C)	BIO7
	Mean temp. wettest quarter (°C)	BIO8
	Mean temp. driest quarter (°C)	BIO9
	Mean temp. warmest quarter (°C)	BIO10
	Mean temp. coldest quarter (°C)	BIO11
Water availability	Annual precipitation (mm)	BIO12
	Precipitation of wettest month (mm)	BIO13
	Precipitation of wettest quarter (mm)	BIO16
	Precipitation of driest quarter (mm)	BIO17
	Precipitation of warmest quarter (mm)	BIO18
	Precipitation of coldest quarter (mm)	BIO19

### 2.2 Bioclimatic modeling of *P. cinereus* between 1961–1980 and 2001–2020

We performed two approaches. We first ran simple models using all predictor variables and three different modeling techniques (glm, gam, and Maxent), to ensure that the choice of technique did not have a noticeable impact on the results. Then, we refined the models made with one of these techniques, namely glm, to conduct all the remaining analyses. First, generalized linear models (glm), generalized additive models (gam), and maximum entropy models (Maxent) were used to assess how bioclimatic variables were correlated with *P. cinereus* presence between 1961–1980 and 2001–2020. Two models were run for each modeling technique, one for each data subset. The predicted function of R was then used to generate presence probabilities across the study region. Correlations among the predictions of all three modeling techniques were high (Table S1, Figures S3 and S4), thus it is unlikely that selecting a particular modeling method would strongly influence the predictions (Appendix S1). Therefore, additional analyses rely on glms, which produce presence probability and can be used to generate favorability (Acevedo & Real, 2012; Real et al., 2006).

To build a more refined glm, we used the multGLM function in the *fuzzySim* R package, using 20% of the data for model testing and 80% for model training for both data sets. This function removes highly correlated variables (among those pairs of variables with a Pearson correlation value above 0.8, it excludes the one with the least informative individual relationship with the distribution of the species). It also removes potentially irrelevant variables following a false discovery rate (Garcia, 2003), and then does a forward‐backward stepwise selection of the remaining variables using an information criterion (AIC; Akaike, 1973; Burnham & Anderson, 2002). Nonsignificant variables after the stepwise procedure were removed using the model Trim function in *fuzzySim* (Crawley, 2007). This demonstrated which variables were significantly correlated with the *P. cinereus* spatial distribution data.

The output of the glm is a presence probability value. Probability depends both on the response of the species to the predictors and on the overall prevalence of the species (Cramer, 1999) (prevalence being the ratio between presences and the total number of cells). To remove the effect of prevalence from the model output, we applied the favorability function proposed by Real et al. (2006):

$$F=\left[P/\left(1-P\right)\right]/\left[\left(n_{1}/n_{0}\right)+\left(P/\left(1-P\right)\right)\right],$$

where *P* is the probability value in a cell, *n*_1_ is the total number of presences, and *n*_0_ is the total number of absences in the data set.

The favorability function reflects the degree (between 0 and 1) to which the local probability values differ from that expected according to the species prevalence, where *F* = 0.5 corresponds to *P* = prevalence. Favorability values only reflect the response of the species to the predictors (Acevedo & Real, 2012) and (unlike probability) can be regarded as the degree of membership of the localities to the fuzzy set of sites with conditions that are favorable for the species (Acevedo & Real, 2012), which enables the easy application of fuzzy logic operations to distribution modeling (e.g., Robertson et al., 2004). Fuzzy logic operations expand the potential of the favorability function for comparison between models for different species, regions, and/or time periods (Estrada et al., 2008, 2011; Real et al., 2010). Favorability was calculated using the Fav function in *fuzzySim*, using the proportion of presences included in the model (training data).

Following Reino et al. (2017), we assessed model performance in terms of discrimination capacity and calibration or reliability for both time periods. The threshMeasures function in the *modEvA* v. 3.5 package (Barbosa et al., 2013) was applied to assess the classification accuracy of the predicted distribution data based on the observed data. This was done using both prevalences as the classification threshold and then again using the maximum true skill statistic (TSS), which optimizes the sum of sensitivity and specificity. Model evaluation metrics (correct classification rate, Sensitivity, Specificity, Precision, Cohen's kappa, and TSS) were calculated using each of these thresholds. Overall model discrimination capacity was assessed using the area under the curve (AUC) of the receiver operating characteristic (ROC) plot. The AUC gives the discrimination performance of the model over the entire range of prediction thresholds. Finally, the correlation between the observations and predictions was tested for significance, and Miller (Miller et al., 1991) calibration statistics were calculated (Wintle et al., 2005).

Favorability has previously been shown to be valuable for comparing species distributions across time periods (Acevedo & Real, 2012; Estrada, Delgado, et al., 2016; Sánchez‐Montes et al., 2019). Differences in favorability for *P. cinereus* were plotted for 1961–1980 and 2001–2020 to compare overall favorability shifts. To assess niche (or potential range) overlap between the two time periods, Schoener's *D* and Warren's *I* (Warren et al., 2008) indices were used. Fuzzy versions (Barbosa, 2015) of similarity indices (Baroni‐Urbani & Buser, 1976; Jaccard, 1901) were also computed using the fuzSim function in *fuzzySim*. Fuzzy range change metrics were assessed between the 1961–1980 and 2001–2020 model predictions, showing fuzzy versions of the overall gain, loss, stability, and balance (the difference between gain and loss) of favorable cells. Two fuzzy logic operations were then applied to the data (expansion, contraction) on the difference between the time periods. In all these fuzzy logic operations, actual favorability values were used with no need to convert predictions to binary before running the operations. This was possible because favorability values are commensurable between species, regions, and/or time periods (see Acevedo & Real, 2012).

### 2.3 Bioclimatic modeling of *P. cinereus* in the future

For future models, we utilized the WorldClim future layers in the *sdmpredictors* package (Hijmans et al., 2005) for the bioclimatic variables included in the modern distribution modeling. This was done for two climate models (CCSM4, MIROC‐ESM), each under four different greenhouse gas (GHG) emissions predictions (representative concentration pathways – RCP 2.6, RCP 4.5, RCP 6.0, RCP 8.5). The same buffered region used for the modern data was used for the future data. The predict function was applied with the refined glm model generated for the 2001–2020 time period. Favorability was assessed for future predictions using the Fav function in the *fuzzySim* package. As above, Schoener's *D*, Warren's *I*, and fuzzy versions of Jaccard's index and Baroni's index were calculated for each GHG emission rate against the 2001–2020 favorability for both future climate models. Similarity indices were also assessed to compare the CCSM4 RCP 8.5 model and the MIROC‐ESM RCP 8.5 model, to assess whether future climate model predictions were more similar than those of the present and future. Fuzzy range change plots were also calculated between the 2001–2020 data and each future model for all four GHG emission rates. Finally, fuzzy logic operations (expansion, contraction) were performed to assess the difference between the present distribution and the most extreme GHG predictions (RCP 8.5) for the CCSM4 and MIROC‐ESM models.

## 3 RESULTS

There was substantial overlap in variables that were included in the glm models for the two time periods (Table 2). In the 1961–1980 model, seven variables were retained (ALT, BIO2, BIO7, BIO8, BIO9, BIO17, and BIO18). In the 2001–2020 model, six variables were retained (BIO7, BIO8, BIO9, BIO16, BIO17, and BIO18). The difference was that ALT and BIO2 were retained in the 1961–1980 model, whereas BIO16 was retained in the 2001–2020 model. Model evaluation measures were then used to determine the fit of each model. For the 1961–1980 model, the classification threshold based on prevalence was 0.024. The model was well supported based on evaluation metrics (CCR = 0.782, sensitivity = 0.827, specificity = 0.780, precision = 0.088, kappa = .119, TSS = 0.607). In the 2001–2020 model, the classification threshold based on prevalence was 0.039. This model was similarly supported (CCR = 0.672, sensitivity = 0.834, specificity = 0.666, precision = 0.092, kappa = .103, TSS = 0.50). Model evaluation metrics were also run using the maximum TSS as a threshold, which generated similar results (Table S2, Figure S5). Further, observations and predictions were significantly correlated with one another for both time periods (*t* = 36.03, df = 146,431, *p* < .001 for 1961–1980 and *t* = 30.91, df = 14,631, *p* < .001 for 2001–2020). The AUC also suggested that the predicted values strongly matched the observed data (AUC = 0.871 for 1961–1980; AUC = 0.827 for 2001–2020). These values indicate a “good” fit (Swets, 1988). Finally, the test data had slopes similar to 1 using the Miller calibration test (*m* = 0.917 for the 1961–1980 test data and *m* = 1.028 for the 2001–2020 data), indicating a good correspondence between predicted probabilities and observed occurrence frequencies.

**TABLE 2:** Results from refined GLM based on *Plethodon cinereus* presences for 1961–1980 and 2001–2020. The sign of the estimate for each variable shows in which direction it influenced presence (e.g., positive estimates imply increased presences associated with the variable). Variable acronyms are outlined in Table 1.

Presence data (1961–1980)				
*P. cinereus* ~ BIO7 + BIO8 + BIO9 + BIO17 + BIO18 + BIO2 + ALT				
	Estimate	Std. error	*z*‐Value	*p*‐Value
Intercept	4.68E+00	5.88E‐01	7.96	<.001
BIO7	−3.18E‐01	1.59E‐02	−19.961	<.001
BIO8	1.50E‐01	6.72E‐03	22.261	<.001
BIO9	−1.42E‐01	6.79E‐03	−20.959	<.001
BIO17	2.89E‐02	1.18E‐03	24.498	<.001
BIO18	−2.28E‐02	1.13E‐03	−20.254	<.001
BIO2	1.54E‐01	3.01E‐02	5.121	<.001
ALT	6.62E‐04	1.55E‐04	4.271	<.001

Presence data (2001–2020)				
*P. cinereus* ~ BIO17 + BIO16 + BIO7 + BIO9 + BIO18 + BIO8				
	Estimate	Std. error	*z*‐Value	*p*‐Value
Intercept	3.19E+00	3.80E‐01	8.40	<.001
BIO17	3.05E‐02	8.93E‐04	34.208	<.001
BIO16	−1.84E‐02	1.19E‐03	−15.434	<.001
BIO7	−1.01E‐01	7.86E‐03	−12.901	<.001
BIO9	−5.77E‐02	4.42E‐03	−13.038	<.001
BIO18	−1.09E‐02	1.34E‐03	−8.118	<.001
BIO8	0.0225351	.0043406	5.192	<.001

**FIGURE 1:**
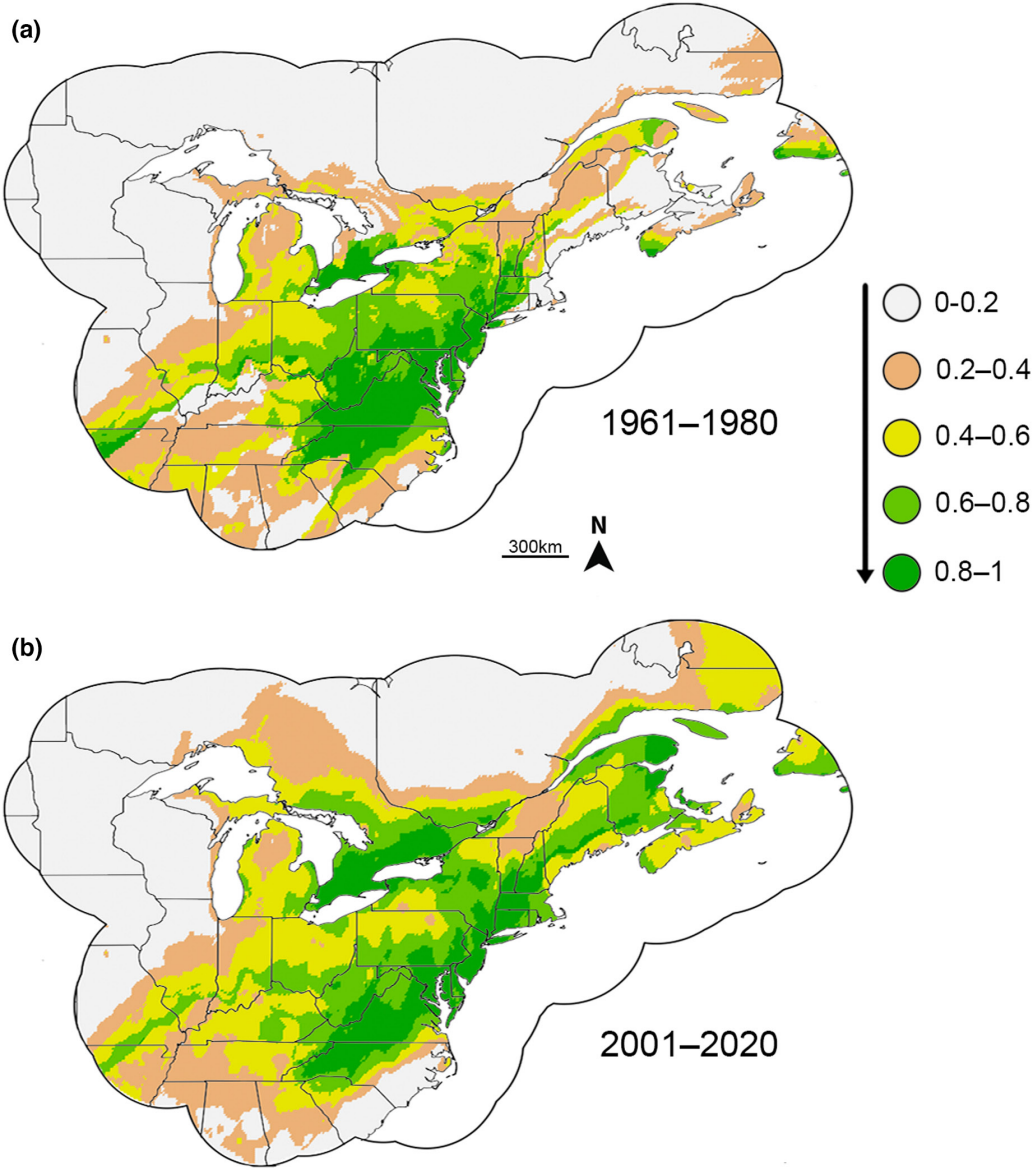
Favorability for *Plethodon cinereus* between (a) 1961–1980 and (b) 2001–2020. Greener colors indicate higher favorability. Note the northward shift in favorability between the two time periods.

**FIGURE 2:**
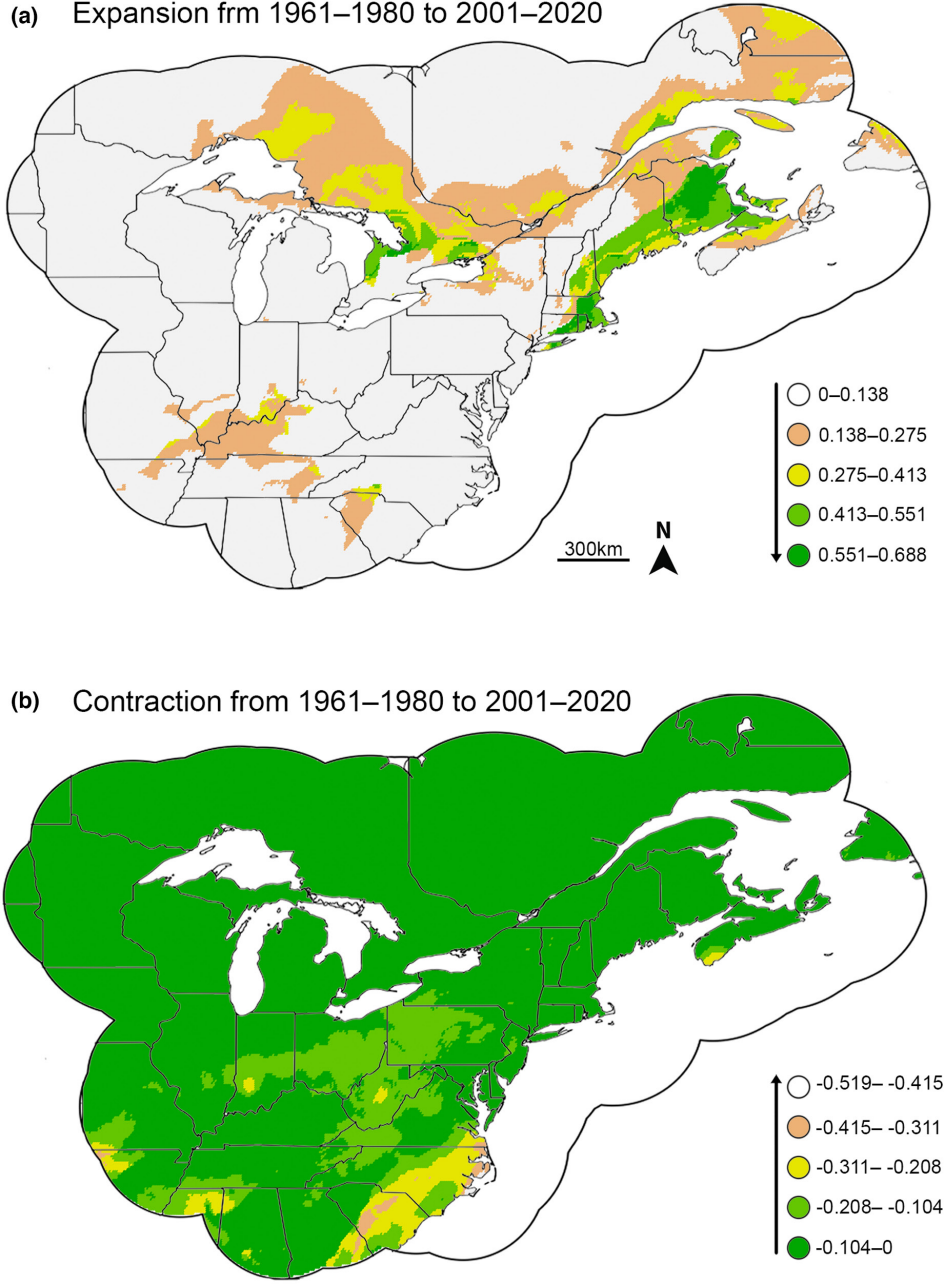
Fuzzy range change for favorability between 1961–1980 and 2001–2020 showing (a) expansion and (b) contraction. For expansion, greener colors show areas of increased expansion, whereas whiter colors do not show areas of substantial expansion. For contraction, whiter colors show areas of increased contraction, whereas greener colors do not show areas of substantial contraction.

Overlap indices showed moderate overlap between the 1961–1980 and 2001–2020 time periods (Schoener's *D* = 0.785; Warren's *I* = 0.959; Jaccard = 0.666, Baroni = 0.835). Fuzzy range change plots showed the most substantial expansion in favorable areas in the northern extreme of the *P. cinereus* range, especially in southern Canada and New England (Figures 1 and 2). There was also moderate expansion of favorability in the western part of the *P. cinereus* range. Favorability contractions were most pronounced on the southeastern part of the *P. cinereus* range, especially in Virginia and the Carolinas. Fuzzy range change metrics suggested substantial gain relative to loss, so the expansion of the favorability range from the 1961–1980 to 2001–2020 time periods was larger than the associated contraction (Table S3).

**FIGURE 3:**
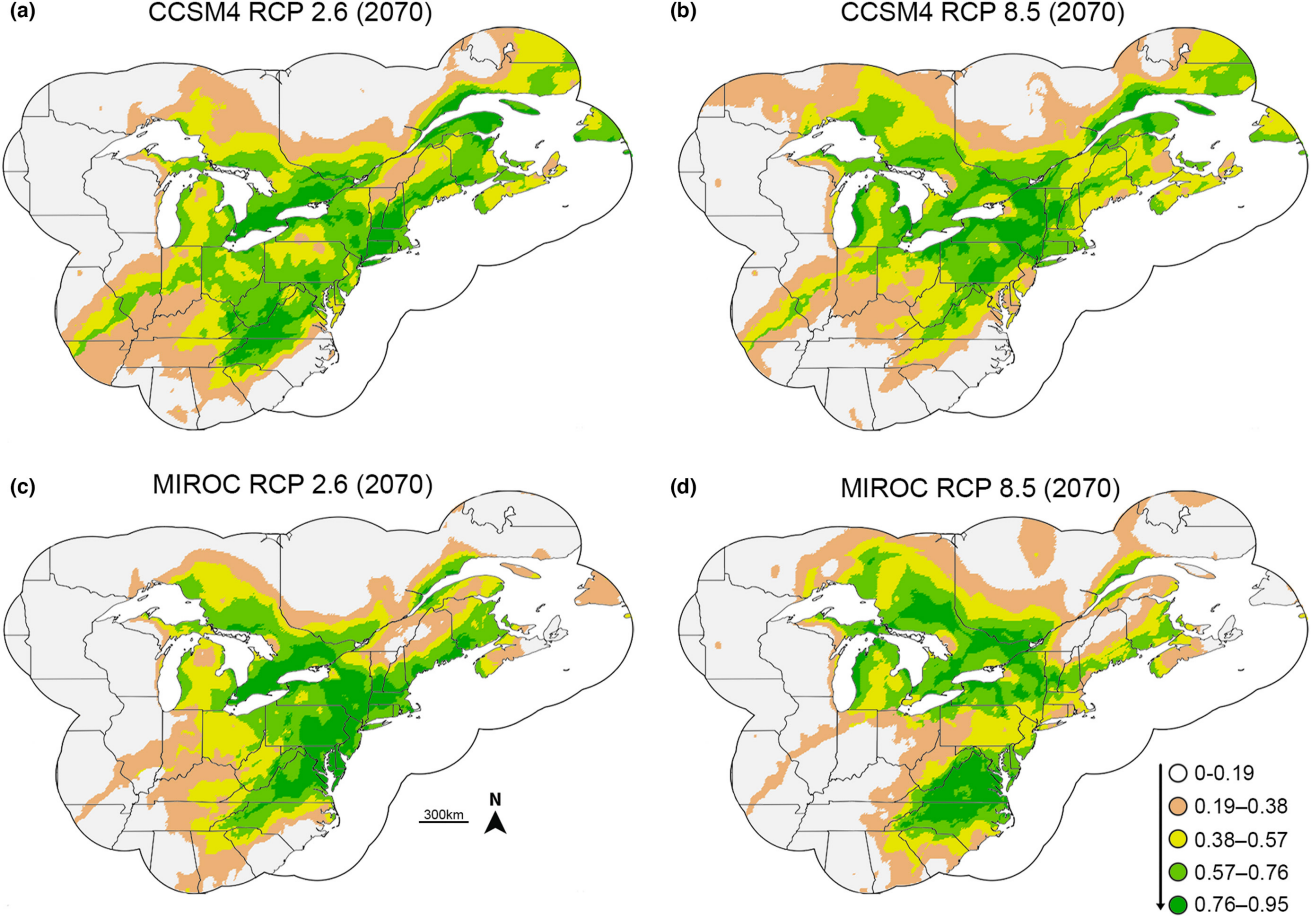
Favorability for *Plethodon cinereus* under the CCSM4 climate model in 2070 for (a) RCP 2.6 and (b) RCP 8.5. Favorability for *P. cinereus* under the MIROC‐ESM climate model in 2070 for (c) RCP 2.6 and (d) RCP 8.5. Note that the northward shift in favorability is more pronounced under higher GHG emissions. The RCP 8.5 model for the MIROC‐ESM climate model also suggests a division between north and south favorability along the mid‐Atlantic region of the United States.

**FIGURE 4:**
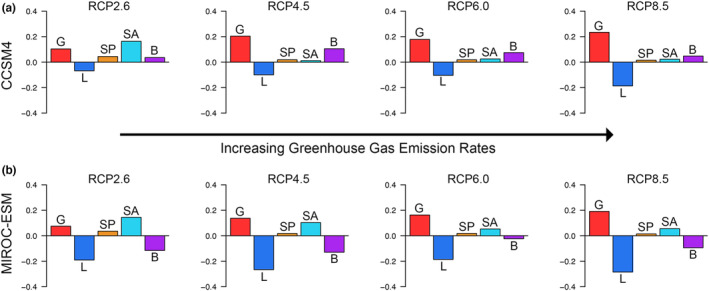
Fuzzy range change metrics for the two climate models (CCSM4, MIROC‐ESM) under four GHG RCPs (2.6, 4.5, 6.0, and 8.5). Each plot shows gained favorability, lost favorability, stable favorability, stable absences, and balance (proportion for gained to lost favorability). Lost favorability generally increases with increasing greenhouse gas emissions.

Both the CCSM4 and MIROC‐ESM climate models point to a potential northward shift in environmental favorability for *P. cinereus* by 2070, with the RCP 8.5 models showing the most substantial northward shift (Figure 3; Figures S6 and S7). Both Schoener's *D* and Warren's *I* indices suggested that predicted GHG pathways of RCP 2.6, 4.5, and 6.0 had a similar amount of overlap with the modern *P. cinereus* favorability range in both climate models. However, RCP 8.5 models yielded a drop in these indices (Table S3). Fuzzy range change metrics for both climate models showed a similar pattern, whereby RCP 2.6 models had a relatively small range change while RCP 4.5 and 6.0 both had higher changes of a similar magnitude, and RCP 8.5 had substantial range gains and losses (Figure 4). Further, across all GHG pathways, the CCSM4 model suggested less change between the present and the future than the MIROC‐ESM model.

Following previous studies, we focused on the RCP 8.5 models for further comparisons (Caravaggi et al., 2017). Northern expansion predictions are similar for the CCSM4 and MIROC‐ESM climate models, with substantial expansion of environmental favorability predicted in southern Canada (Figure S8). The CCSM4 model predicts more substantial favorability range expansion than the MIROC‐ESM model north of the modern *P. cinereus* range. The MIROC‐ESM model predicts additional range expansion south of the current *P. cinereus* range in North Carolina, which is not supported under the CCSM4 model. Both models predict contractions in the southern part of the modern *P. cinereus* range, particularly in New Jersey and the Midwest (Ohio, Indiana, and Illinois) (Figure S8). The CCSM4 and MIROC‐ESM models, however, differ in that the contraction in the CCSM4 model implies contraction in favorability throughout the modern southern *P. cinereus* range (including Mid‐Atlantic states such as Maryland, Virginia, North Carolina in addition to the aforementioned midwestern states). The MIROC‐ESM model predicts additional favorability range contraction further north than the CCSM4, insinuating potential fragmentation of the modern *P. cinereus* favorable region into northern and southern favorable regions, with contractions in the northeastern states of the US, and even southeastern Canada.

## 4 DISCUSSION

Climate change is likely to cause major shifts in environmental suitability for a wide range of taxa, which will undoubtedly lead to range shifts and local extirpations (Pacifici et al., 2015; Van der Putten, 2012). Dispersal‐limited species are likely to be more strongly impacted by these shifts because they cannot quickly respond to rapid environmental change. Given the wide geographical range of *P. cinereus* and the numerous investigations into its life history and behavior (reviewed in Fisher‐Reid et al., 2021), as well as the importance of salamanders generally as environmental indicators (Buckley & Jetz, 2007; Feder & Burggren, 1992; Fleming et al., 2020; Welsh & Droege, 2001), this species is an excellent model system for utilizing species distribution models/ecological niche models to better understand the macroclimatic factors driving past, present, and future potential range shifts in dispersal‐limited amphibians.

We found that a combination of precipitation and temperature variables define the regions that are currently climatically favorable for *P. cinereus*. In particular, regions with increased *P. cinereus* environmental favorability are more strongly associated with temperate, milder climates. Additionally, we found a northward shift in favorability between 1961–1980 and 2001–2020. Climate models indicate an additional northward shift by 2070. Given the low dispersal rates of *P. cinereus* and amphibians generally (Abellán & Svenning, 2014; Muñoz, Miller Hesed, et al., 2016; Recuero & García‐París, 2011), this may suggest future extirpations.

### 4.1 The impact of bioclimatic variables on *P. cinereus'* distribution

Due to their reliance on water for breeding, amphibians have been shown to be tied strongly to precipitation‐based climate variables (Aragón et al., 2010; Buckley & Jetz, 2007; Duellman, 1999). Since most amphibians lay eggs in water, particularly in vernal pools, variation in precipitation can strongly impact amphibian reproductive success (Carey & Alexander, 2003). For example, variation in rainfall can change the number of eggs laid in a particular year (Caldwell, 1987). Although plethodontids are not tied to water for breeding, adult survivorship is also impacted by precipitation since they respire through their skin and have relatively high rates of water loss (Carey & Alexander, 2003; Lunghi, Manenti, et al., 2018; Shoemaker et al., 1992). Plethodontids lay their eggs in moist areas under rocks or fallen trees, with some species utilizing subterranean environments (Lunghi, Corti, et al., 2018), and they spend time at the surface to feed (Petranka, 1998). As a result, *P. cinereus* must spend time at the surface and, given their physiological limitations, increased precipitation and soil moisture grant them the necessary conditions for surface activity (Gade & Peterman, 2019; Milanovich et al., 2010; Wilk et al., 2020). Further, experimental analyses have shown that increased temperatures reduce growth rates in *P. cinereus* (Muñoz, Miller Hesed, et al., 2016). Higher mean maximum July temperatures and lower precipitation in the driest month has been shown to be correlated with a 1.8% increase in *P. cinereus* body size between 1950–1970 and 1980–2000 (McCarthy et al., 2017). Therefore, changes to both precipitation and temperature variables are likely to strongly impact *P. cinereus* physiology.

Our models suggest that favorable regions for *P. cinereus* in the present (2001–2020) are centered on climatic stability (Table 2). *P. cinereus* presence was correlated with higher precipitation in the driest quarter (*BIO17*), lower precipitation in the wettest quarter (*BIO16*), lower annual temperature range (*BIO7*), lower temperature in the driest quarter (*BIO9*), lower precipitation in the warmest quarter (*BIO18*), and higher mean temperature in the wettest quarter (*BIO8*). These factors combined do not suggest that higher precipitation implies the higher chance of presence, but rather that moderate, mild climates lead to higher presence probability and more climatically favorable regions for *P. cinereus*. This may relate to a wider pattern of niche conservatism in climatic tolerances found in plethodontids in Appalachia, whereby the highest species diversity is found in intermediate‐elevation habitats (Kozak & Wiens, 2010). Kozak and Wiens (2010) suggest that plethodontids are specialized to a certain set of environmental conditions leading to a build‐up of species in regions mimicking their ancestral environments due to niche conservatism. Climate change is leading to a greater frequency and severity of both precipitation and temperature extremes (Stott, 2016; Zhang et al., 2013). Therefore, this has strong implications for *P. cinereus* distributions in the future.

### 4.2 Environmental favorability range shifts in the past, present, and future

Our data document a clear northward shift in environmental favorability between 1961–1980 and 2001–2020. When viewing the presence data for 1961–1980 and 2001–2020, it is evident that the center of the distribution has moved from an area ranging from southern Virginia to central Ohio (Figure S1b) north to central Virginia to southern Canada (Figure S1c). Expansion of environmental favorability occurred in the northern part of the range and contraction occurred in the southern part between these time periods (Figure 2). Although the regional shift is clear, models suggested a similar set of bioclimatic factors explaining presence for both time periods (Table 2). These past and recent data serve as a baseline for our analyses using future climate models and provide a clear pattern showing the northward shift in environmental favorability for *P. cinereus* in the recent past.

Although our data supported the prediction that favorable regions would shift north between 2001–2020 and 2070 and that this would be most extreme under increasing GHG emissions models, we also found that gained favorable areas increased with increasing GHG emissions, contrary to our expectations (Figure 4). Similarly, lost favorable areas increased as GHG emissions rates increased, suggesting the strongest range shifts under higher GHG emissions, similar to what has been found in previous studies examining favorability shifts as a result of increasing GHG emissions (Báez et al., 2020; Morales‐Castilla et al., 2020). Lost favorable areas were generally in the southern part of the range (especially in the CCSM4 model) while gained favorable areas were generally in the northern part of the range (Figure S8). This pattern mimicked the trend found between 1961–1980 and 2001–2020. Despite these model‐based trends in environmental favorability, recent field‐based work has demonstrated that a population of *P. cinereus* in the southern edge of their range in Virginia has higher densities and larger home ranges relative to more northern sites in Pennsylvania and New York (Hernández‐Pacheco et al., 2019). Therefore, it is possible that other factors such as phenotypic plasticity are mediating larger‐scale climate effects on *P. cinereus*. However, *P. cinereus* is generally poorly sampled at the extremes of its range compared to the core of its range, and additional long‐term field studies are necessary to confirm the generalizability of the sites in Virginia across the southern range and to document possible changes in the next few decades along the southern extreme. Such studies would provide a window into possible transitions between local to dark diversity (Pärtel et al., 2011) for *P. cinereus* and would inform future SDMs.

Since *P. cinereus* spends most of its life underground, soil temperature 30 cm below the surface (rather than air temperature or precipitation) serves as the best metric for predicting detection rates (Sanchez et al., 2020)*. P. cinereus* are likely able to modulate their position in the soil column so that they are at optimum environmental conditions (Muñoz, Miller Hesed, et al., 2016). However, *P. cinereus* does need to come to the surface to feed and mate (Houck & Verrell, 1993; Tilley & Bernardo, 1993). More variable macroclimate conditions will likely have strong effects on favorable surface conditions for salamanders, and in turn impact their behavior and physiology (Muñoz, Miller Hesed, et al., 2016). Warmer temperatures have been shown to negatively impact autumn growth in *P. cinereus*, increasing the time until individuals are sexually mature (Muñoz, Miller Hesed, et al., 2016). Surface activity is impacted partly by macroclimatic variables, but also by microclimatic fluctuations. For example, canopies buffer against microclimatic variability (Chen et al., 1999), and therefore, canopy loss may have strong local effects on salamanders (Demaynadier & Hunter, 1998; Harpole & Haas, 1999).

Many salamander species, including *P. cinereus*, have generally small home ranges and low dispersal capabilities (Abellán & Svenning, 2014; Lunghi & Bruni, 2018; Muñoz, Miller Hesed, et al., 2016; Recuero & García‐París, 2011). Indeed, previous work has shown that *P. cinereus* individuals are usually found within a meter of their original capture site (Gergits & Jaeger, 1990). Given that individuals spend a substantial component of their lifetime underground, imperfect detection may impact our understanding of environmental preferences generated through presence data. Using models that account for detection variability through repeated site visits (e.g., site occupancy models) could improve the robusticity of environmental favorability projections. However, the available species data make it impossible to run these types of analyses at this spatial scale. Further, while climatic variables typically play a large role in determining large‐scale distribution patterns with land use and human‐related variables often acting at finer scales, anthropogenic variables certainly have some explanatory value in describing salamander occurrence data. However, since a major component of our work was to evaluate favorability shifts in the future, and reliable predictions of human variables by 2070 would strongly increase uncertainty in our projections, we only examine elevation and climate here. Finally, species dispersal capabilities are a critical component to realizing range shifts (Kearney & Porter, 2009; Thomas et al., 2004) and low dispersal capabilities may lead to results that conflict with distribution model predictions (Pearson & Dawson, 2003). As a result, northward shifts in environmental favorability for *P. cinereus* do not necessarily imply northward shifts in the species' distribution range. Our data may suggest extirpation (if no local adaptation occurs) in the southern regions as they become less environmentally favorable, without the coincident northward range expansion implied by our models (Figure 3). Similarly, these models are correlative and do not include the fact that *P. cinereus* may modify its behavior or be physiologically plastic to persist in environmentally unfavorable conditions at the southern edge of its range (Newman et al., 2022; Riddell & Sears, 2020). As a result of these factors, the shifts in favorability should be best viewed as potentially suitable areas and future work may improve our projections with additional analyses.

On the contrary, other amphibian distribution modeling work has found that *Lissotriton helveticus* was able to shift northward during rapid warming in the Holocene despite low dispersal capabilities (Recuero & García‐París, 2011). Other amphibians such as *Hyla molleri* were not strongly impacted by climate fluctuations since the Last Interglacial, since it has a relatively high cold tolerance (Sánchez‐Montes et al., 2019). Further, recent work has shown that the majority of *P. cinereus* genetic diversity is in the southern part of its modern range (Radomski et al., 2020). Three quarters of the modern *P. cinereus* range was covered by glaciers during the last glacial maximum (LGM) and as such, rapid range expansion after the LGM is postulated (Radomski et al., 2020). *Plethodon albagula* was shown to have relatively low gene flow through favorable habitats, suggesting that plethodontids may move quickly and directly through inhospitable habitats while they move in a slower, more exploratory fashion in favorable habitats (Peterman et al., 2014), potentially increasing dispersal capabilities when pressed. Thus, *P. cinereus* and other amphibians may have higher dispersal capabilities than currently realized. Further, the large population size of *P. cinereus* may enhance the likelihood of persistence and future recolonization from climate refugia following rapid climatic shifts (Recuero & García‐París, 2011). It is noteworthy that climate is often found to be less impactful on species than direct anthropogenic changes (Gutiérrez‐Rodríguez et al., 2017b; Hampe & Jump, 2011). Matching SDM and phylogeographic data with dispersal simulations (e.g., Pearson & Dawson, 2003; Peterson et al., 2001) would be a fruitful avenue for better understanding how dispersal‐limited species will react to future shifts in climate favorability.

## 5 CONCLUSION

Dispersal‐limited species, such as salamanders, are particularly susceptible to rapid changes in environmental favorability as a result of climate change (Chevin et al., 2010; Midgley et al., 2006; Muñoz, Miller Hesed, et al., 2016). Our modeling suggests that the favorable environmental conditions for *P. cinereus* have shifted northward between 1961–1980 and 2001–2020 (Figures 1 and 2), likely as a result of climate change. Further northward shifts in favorability are predicted using two climate models (CCSM4, MIROC‐ESM), with gains in favorability largely in the northern extent of the current range and losses in the southern part of the range. Species distribution models/ecological niche models provide a foundation for where we should expect to lose *P. cinereus* populations using macroclimatic variables. To better understand the effects of microclimatic changes on *P. cinereus* and other salamanders accompanying climate change, as well as determine whether the decreases in favorability predicted by our models are fulfilled, more long‐term field studies are necessary (Fisher‐Reid et al., 2021), especially at the extremes of the distribution range. Combining species distribution models using macroclimatic variables with field data from local, finer‐scale variables (Enriquez‐Urzelai, Kearney, et al., 2019) will help to elucidate future trends for *P. cinereus*, for North American salamanders, and for dispersal‐limited taxa more generally.

## AUTHOR CONTRIBUTIONS

**Brandon P. Hedrick:** Conceptualization (lead); data curation (lead); formal analysis (lead); methodology (equal); writing – original draft (lead); writing – review and editing (equal). **Alba Estrada:** Conceptualization (supporting); formal analysis (supporting); methodology (equal); supervision (equal); writing – original draft (supporting); writing – review and editing (equal). **Chris Sutherland:** Conceptualization (supporting); methodology (equal); supervision (equal); writing – original draft (supporting); writing – review and editing (equal). **A. Márcia Barbosa:** Conceptualization (supporting); formal analysis (supporting); methodology (equal); supervision (equal); writing – original draft (supporting); writing – review and editing (equal).

## CONFLICT OF INTEREST STATEMENT

The authors declare that they have no conflict of interest.

## Supporting information


Figure S1

Figure S2

Figure S3

Figure S4

Figure S5

Figure S6

Figure S7

Figure S8

Tables S1–S3

Appendix S1

## Data Availability

Data required to replicate analyses are available in the [Link], [Link], [Link], [Link], [Link], [Link], [Link], [Link], [Link], [Link] of the manuscript and from GBIF via https://doi.org/10.15468/dl.d3j5ys. Code is available at https://github.com/bphedrick/E‐E‐Salamander‐Ecology.
