# Supplementary figures and images for: Projected northward shifts in eastern red‐backed salamanders due to changing climate
Source: Ecol Evol. 2023 Apr 26;13(4):e9999. doi: 10.1002/ece3.9999 (PMC10133384; doi:10.1002/ece3.9999)

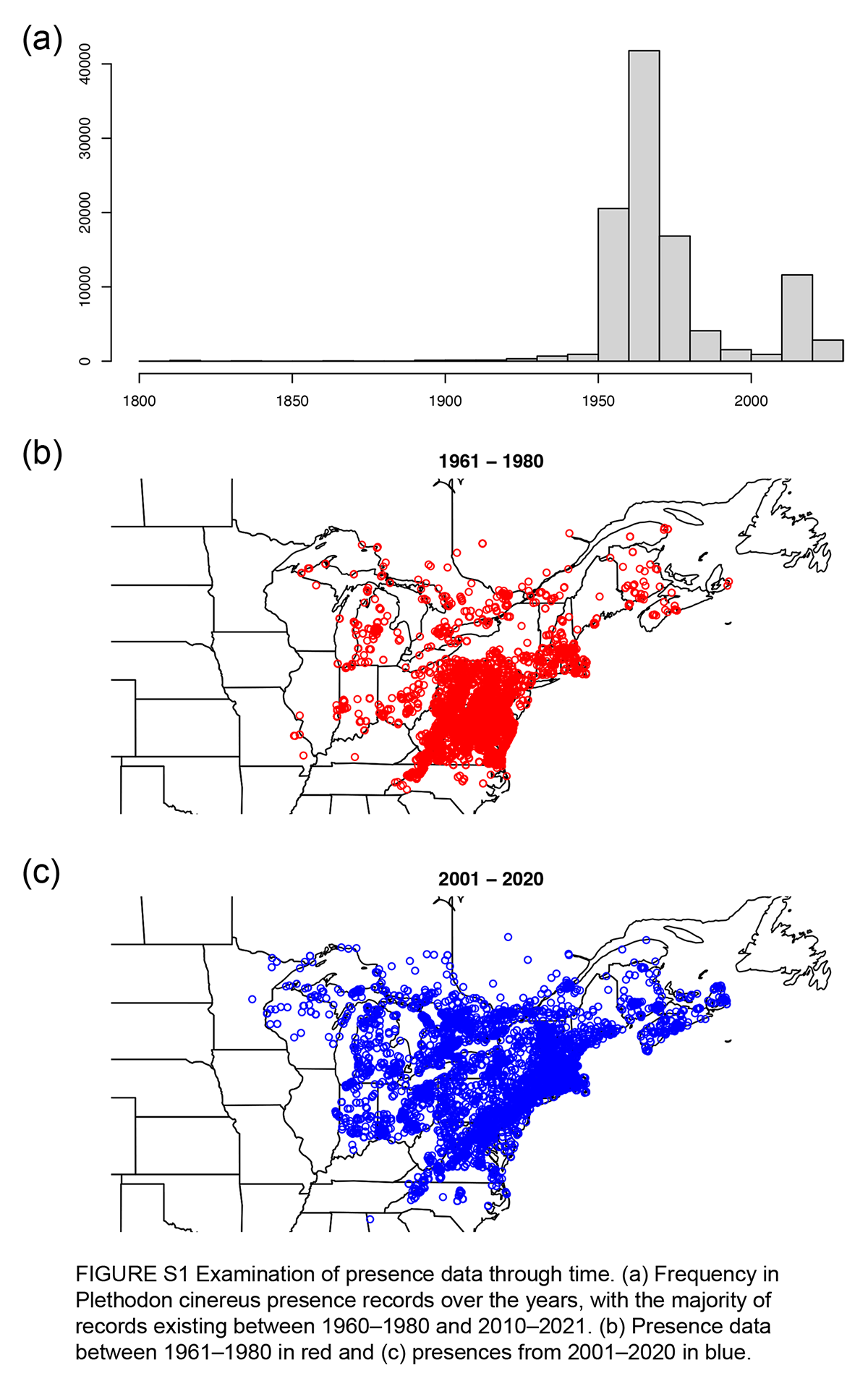

Supplement: Supplementary file 1 — Figure S1 [file ECE3-13-e9999-s006.tif]

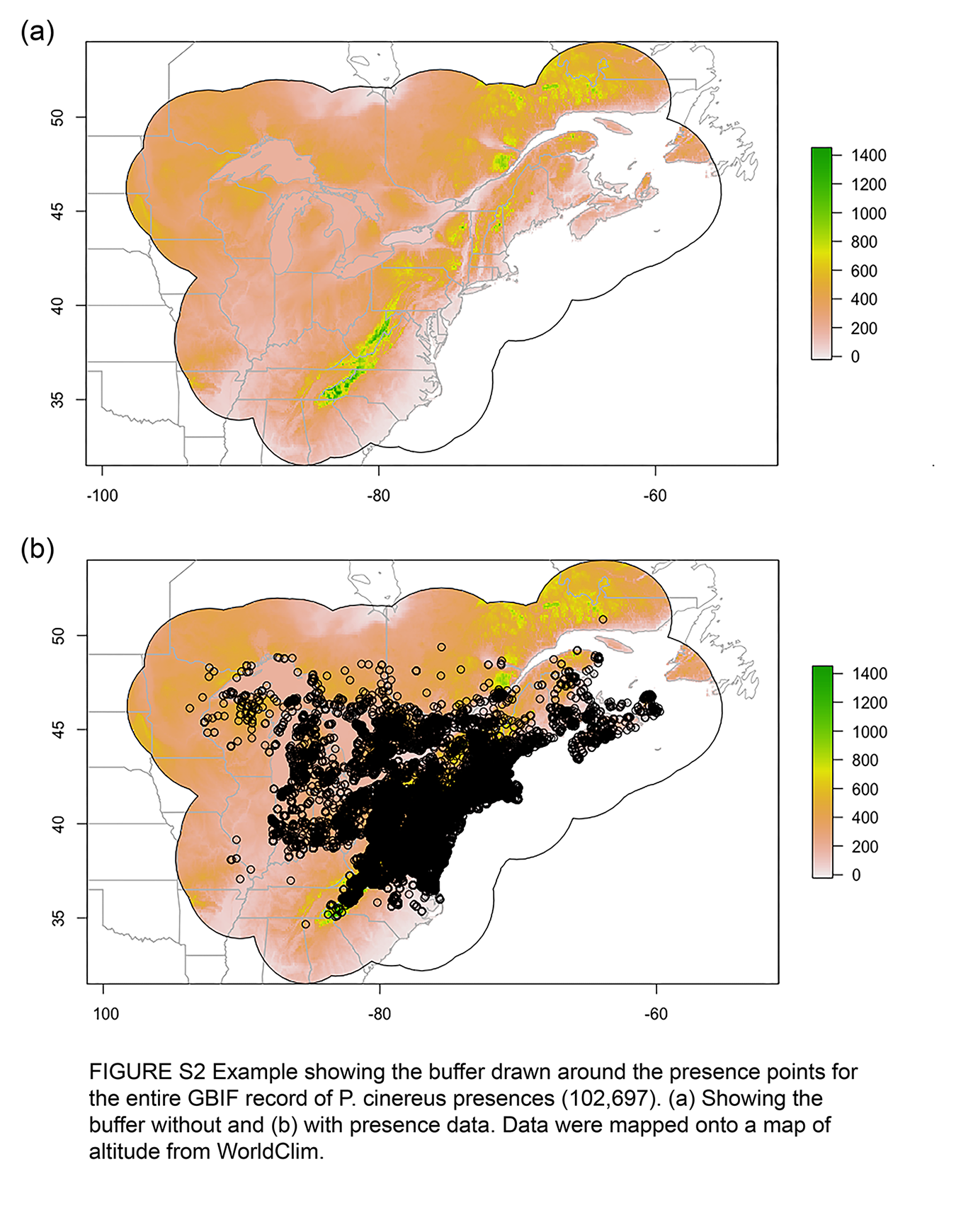

Supplement: Supplementary file 2 — Figure S2 [file ECE3-13-e9999-s005.tif]

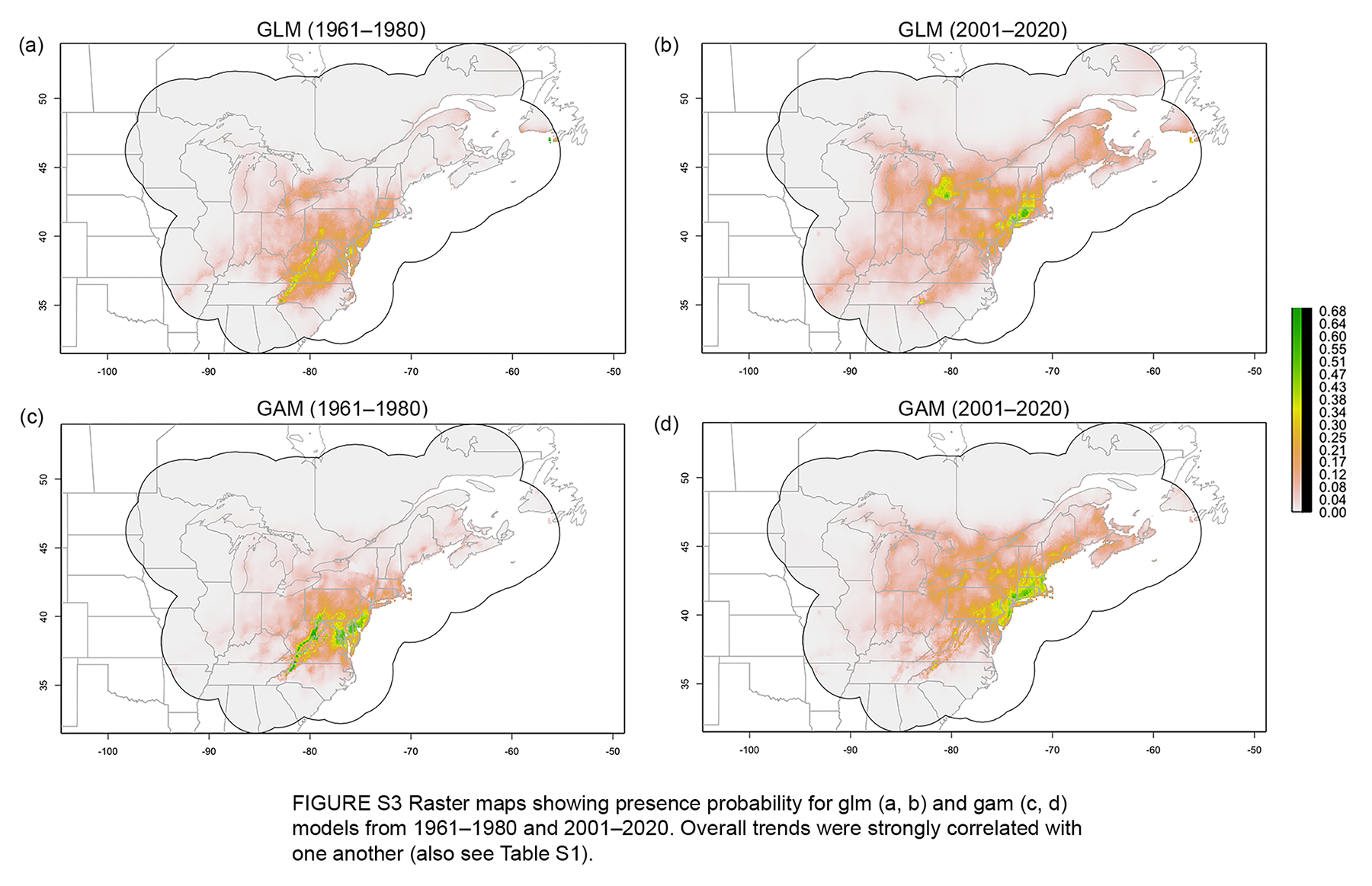

Supplement: Supplementary file 3 — Figure S3 [file ECE3-13-e9999-s003.tif]

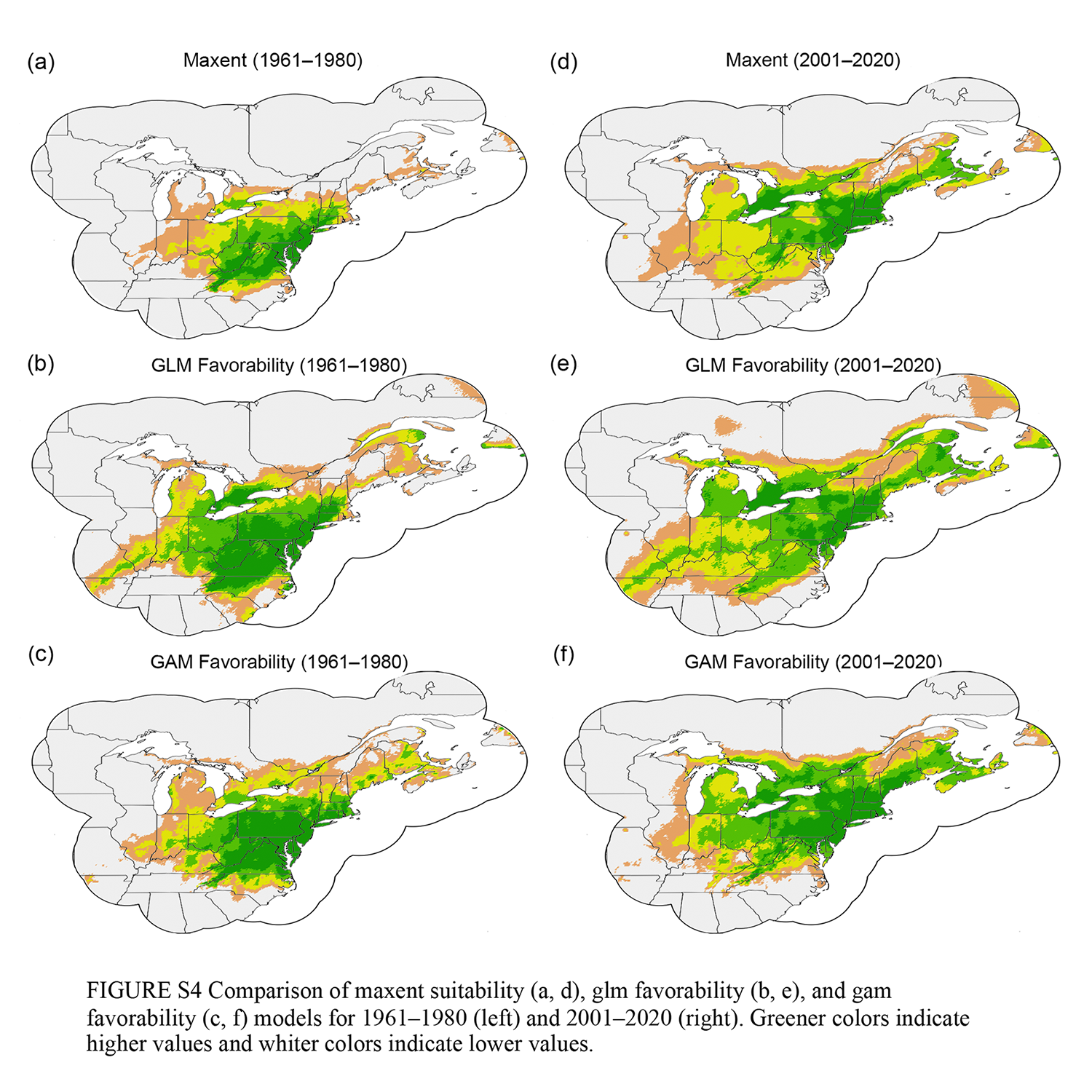

Supplement: Supplementary file 4 — Figure S4 [file ECE3-13-e9999-s001.tif]

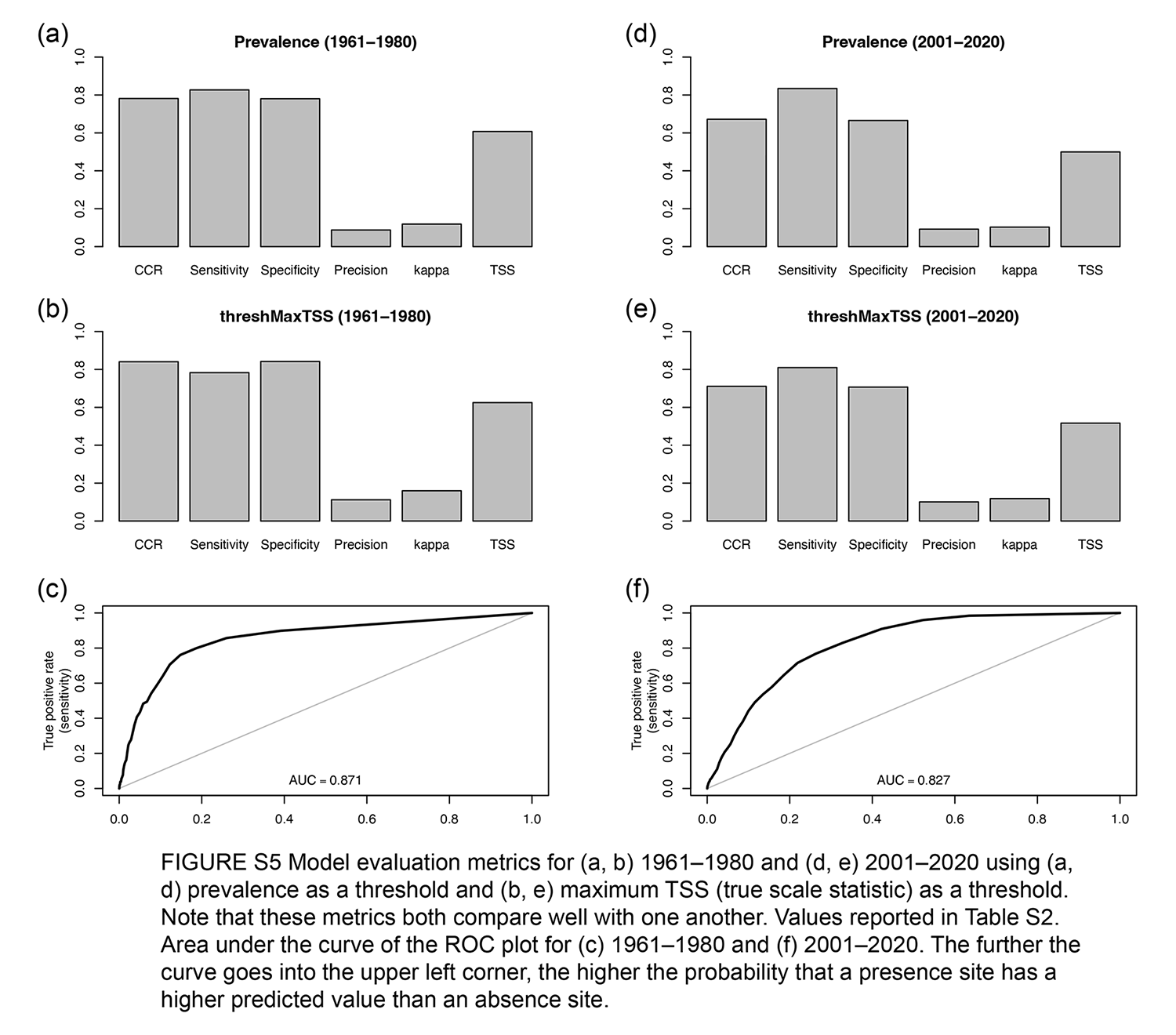

Supplement: Supplementary file 5 — Figure S5 [file ECE3-13-e9999-s004.tif]

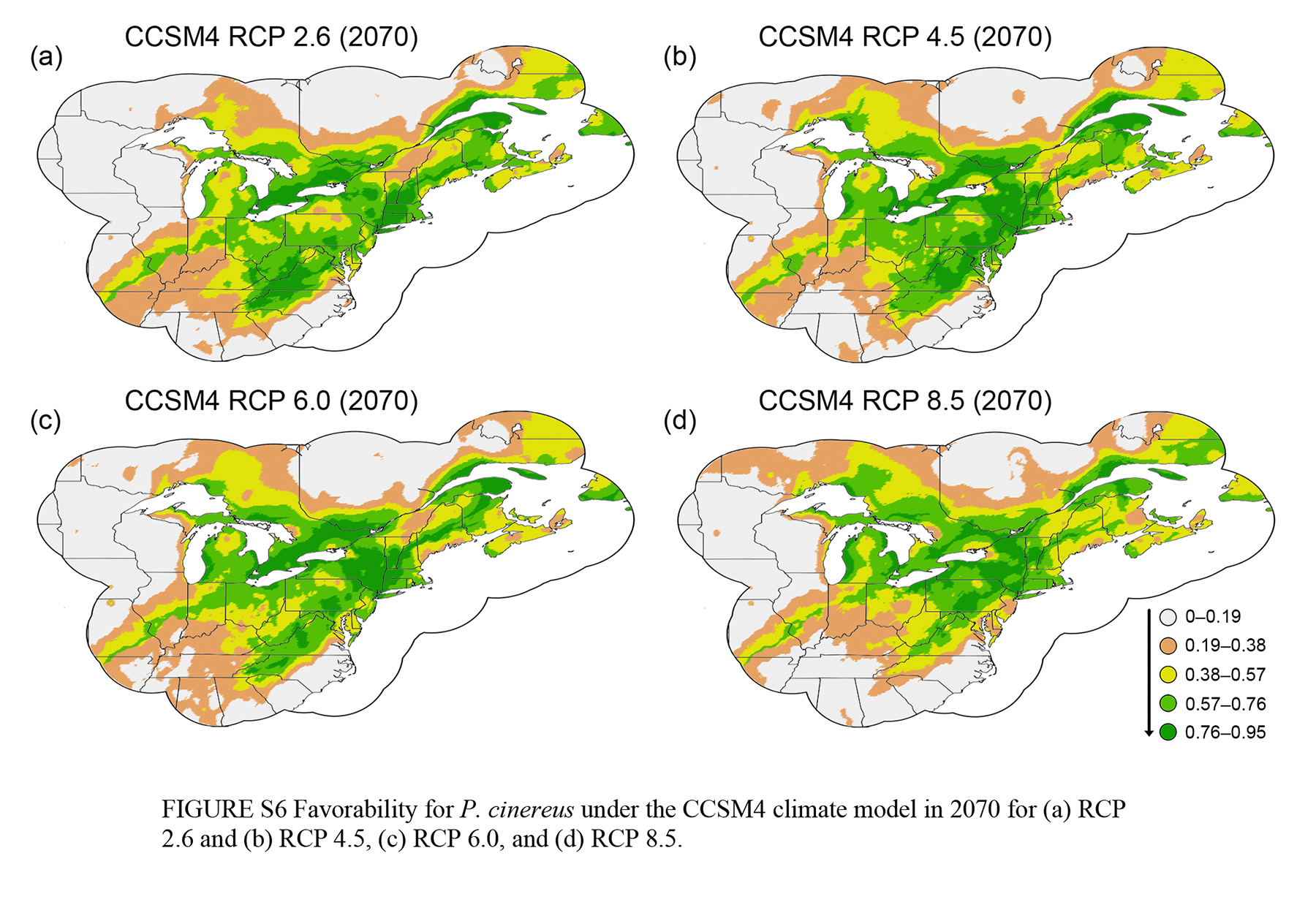

Supplement: Supplementary file 6 — Figure S6 [file ECE3-13-e9999-s008.tif]

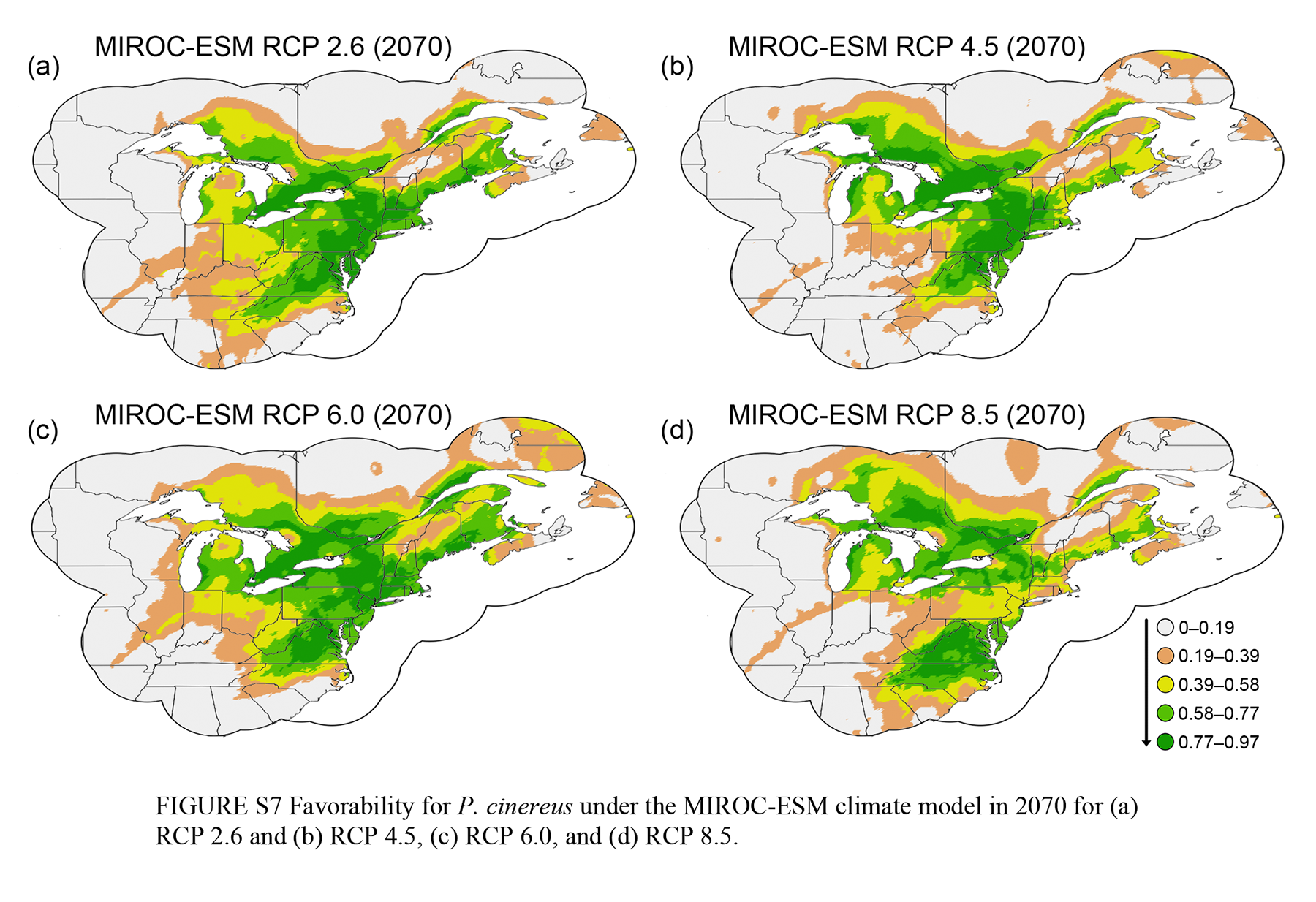

Supplement: Supplementary file 7 — Figure S7 [file ECE3-13-e9999-s002.tif]

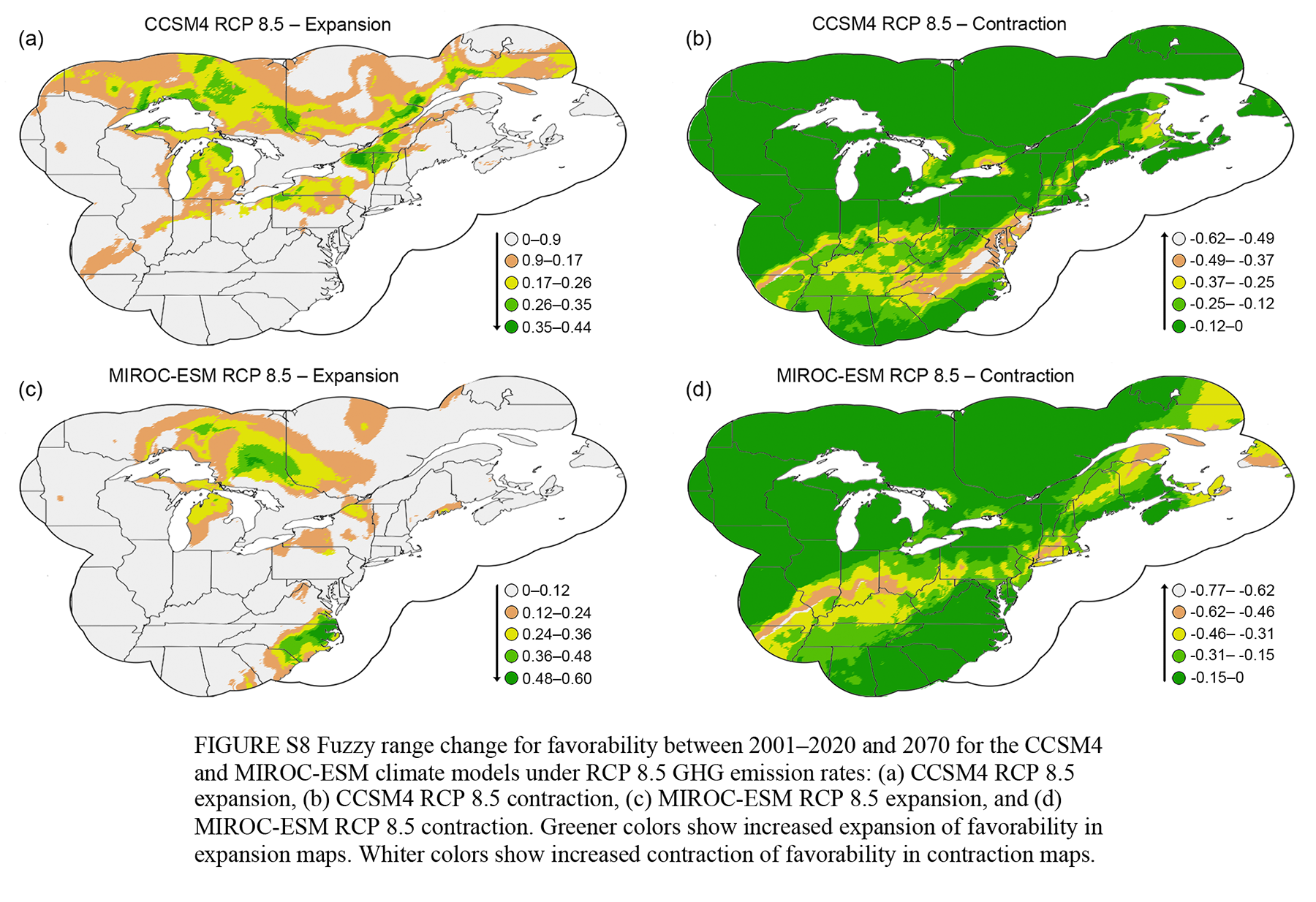

Supplement: Supplementary file 8 — Figure S8 [file ECE3-13-e9999-s007.tif]
