# Supplementary material for: Projected northward shifts in eastern red‐backed salamanders due to changing climate
Source: Ecol Evol. 2023 Apr 26;13(4):e9999. doi: 10.1002/ece3.9999 (PMC10133384; doi:10.1002/ece3.9999)
Supplement: Supplementary file 10 — Appendix S1 [file ECE3-13-e9999-s010.docx]

**Supplemental Information:**

To assess how different models compared when assessing *P. cinereus* presence between 1961–1980 and 2001–2020, three models were used: generalized linear models (glm), generalized additive models (gam), and maximum entropy models (Maxent). As with the glm models in the main text, each model was run once using the presence data from 1961–1980 and again with the presence data from 2001–2020. The gam model was run under the same parameters as the glm model (presence ~ altitude and 16 bioclimatic variables) but using smoothing splines for each variable. Gam models were then projected onto raster maps to compare directly with glm model projections (Figure S3). Both these models generate presence probability for each map pixel.

Maxent models are presence-background models (which generate environmental suitability) and therefore, the presence-absence data used in the glm and gam models were converted to presence (1) and background (0) data in the Maxent model, whereby presences were also included in the background. The maxnet function was used in the *maxnet* package v. 0.1.4 (Phillips, 2017) with feature classes set to linear and quadratic. Predictions were calculated using the predict function with the complementary log-log (cloglog) link function. These predictions were then projected onto the raster maps of the buffered region to show differences between maxent predictions and favorability from glm and gam models (Figure S4).

Correlations between model predictions were then calculated and plotted to assess how all three models compared to one another (Table S1). Generally, model correlations were high for presences (minimum correlation for 1961–1980 = 0.813; minimum correlation for 2001–2020 = 0.827). They were similarly high for favorability (minimum correlation for 1961–1980 = 0.895; minimum correlation for 2001–2020 = 0.874). The glm and gam raster map predictions based on presences were very similar to one another (Figure S3), as were glm and gam maps based on favorability when compared with maxent maps (Figure S4). Given that correlations are high, it is unlikely that selecting a particular model will strongly influence predictions. Therefore, we opted to present results in the main text based on favorability derived from the glm model.

Code associated with this project for the replication of analyses is available at: https://github.com/bphedrick/E-E-Salamander-Ecology

FIGURE S1 Examination of presence data through time. (a) Frequency in *Plethodon cinereus* presence records over the years, with the majority of records existing between 1960–1980 and 2010–2021. (b) Presence data between 1961–1980 in red and (c) presences from 2001–2020 in blue.

FIGURE S2 Example showing the buffer drawn around the presence points for the entire GBIF record of *P. cinereus* presences (102,697). (a) Showing the buffer without and (b) with presence data. Data were mapped onto a map of altitude from WorldClim.

FIGURE S3 Raster maps showing presence probability for glm (a, b) and gam (c, d) models from 1961–1980 and 2001–2020. Overall trends were strongly correlated with one another (also see Table S1).

FIGURE S4 Comparison of maxent suitability (a, d), glm favorability (b, e), and gam favorability (c, f) models for 1961–1980 (left) and 2001–2020 (right). Greener colors indicate higher values and whiter colors indicate lower values.

FIGURE S5 Model evaluation metrics for (a, b) 1961–1980 and (d, e) 2001–2020 using (a, d) prevalence as a threshold and (b, e) maximum TSS (true scale statistic) as a threshold. Note that these metrics both compare well with one another. Values reported in Table S2. Area under the curve of the ROC plot for (c) 1961–1980 and (f) 2001–2020. The further the curve goes into the upper left corner, the higher the probability that a presence site has a higher predicted value than an absence site.

FIGURE S6 Favorability for *P. cinereus* under the CCSM4 climate model in 2070 for (a) RCP 2.6 and (b) RCP 4.5, (c) RCP 6.0, and (d) RCP 8.5.

FIGURE S7 Favorability for *P. cinereus* under the MIROC-ESM climate model in 2070 for (a) RCP 2.6 and (b) RCP 4.5, (c) RCP 6.0, and (d) RCP 8.5.

FIGURE S8 Fuzzy range change for favorability between 2001–2020 and 2070 for the CCSM4 and MIROC-ESM climate models under RCP 8.5 GHG emission rates: (a) CCSM4 RCP 8.5 expansion, (b) CCSM4 RCP 8.5 contraction, (c) MIROC-ESM RCP 8.5 expansion, and (d) MIROC-ESM RCP 8.5 contraction. Greener colors show increased expansion of favorability in expansion maps. Whiter colors show increased contraction of favorability in contraction maps.

TABLE S1 Model correlations for presence data across GLM, GAM, and Maxent models (top table). Model correlations between GLM favorability, GAM favorability, and Maxent suitability (bottom table). 1961–1980 above diagonal and 2001–2020 below diagonal in both tables. Reported values are Pearson correlation coefficients.

TABLE S2 Model evaluation metrics (CCR, sensitivity, specificity, precision, kappa, and TSS) for 1961–1980 and 2001–2020. Evaluation metrics were calculated first using prevalence as a threshold and then using maximum TSS (true skill statistic) as a threshold for each time period. Pseudo-R^2^ metrics are also reported for each time period.

TABLE S3 Model evaluation metrics between 1961–1980 and 2001–2020 as well as 2001–2020 and 2070 under two climate models (CCSM4, MIROC-ESM) and four representative concentration pathways (2.6, 4.5, 6.0, and 8.5). Fuzzy range change metrics for comparisons between 1961–1980 and 2001–2020 as well as between 2001–2020 and future models. Finally, model evaluation metrics were calculated between the two future models under RCP 8.5.
